# The Creative Process and Emotions of Pupils in a Training Context with a Design Project

**DOI:** 10.3390/jintelligence10040108

**Published:** 2022-11-17

**Authors:** Marion Botella, John Didier, Marie-Dominique Lambert, Rachel Attanasio

**Affiliations:** 1 Université Paris Cité and Univ Gustave Eiffel, LaPEA, F-92100 Boulogne-Billancourt, France; 2 Haute Ecole Pédagogique, Education and Research Art and Technology Unit, 1014 Lausanne, Vaud, Switzerland

**Keywords:** creative process, pupils, multivariate factors, Creative process Report Diary, design-activity

## Abstract

For many years, researchers have been investigating how the creative process occurs and what factors influence it. The scope of these studies is essential in the school context to enable pupils to develop their creativity and thus address the needs of the 21st century society. Although very rich, these studies are generally not situated in a real teaching and learning context. The output of the present research will make it possible to model, to better understand, and to identify the creative process in pupils as they design and produce utility objects in an educational and training context with ecological validity (real context of training). In the context of teaching Creative and Manual Activities in education, in the French part of Switzerland, we are focusing on observations of the creative process in line with psychology, didactics, and pedagogy. During their class, 22 pupils were invited to create a water fountain and, in parallel, to complete a Creative process Report Diary about the stages they do and the multivariate factors (cognitive, conative, emotional, and environmental factors) they mobilize at each lesson. Results presented the main frequent stages and factors at each lesson and we proposed a model describing the transitions between the stages and how the multivariate factors are involved in each stage. They illustrate what pupils actually do in a creative learning context.

## 1. Introduction

For around thirty years, creativity has been defined as the capacity to produce a novel, original idea that fits with task constraints (Lubart 1994; Runco and Jaeger 2012). This idea must be novel in the sense that it goes beyond a replication or copy of that which exists. This capacity to generate creative productions serves as an economic and social catalyst for society. A creative experience is indeed an unexpected encounter between a person and the world that involves commitment on his or her part (Glăveanu and Beghetto 2021). Today, creativity is a major asset for our society; for instance, the strength of our economy is mainly based on new and “good” ideas (i.e., creative ideas) and not so much on our production capacity (Miller and Dumford 2014; Tepper and Kuh 2011). However, even now, the creative process of transforming creativity into objects is still enigmatic. This effect could be due to the difficulty for creators to describe their own process experiences. The creative process could be more enigmatic for pupils who have not experienced it frequently before now. In the following sections, studies describing the creative process will be presented with particular attention to those conducted in the field of design, and then the types of factors that influence the creative process will be described. Finally, the process and the factors will be contextualized to the teaching of creativity in schools and more specifically in the Creative and Manual Activities courses in Switzerland.

### 1.1. The Creative Process

Based on introspective accounts of eminent creators (such as artists and scientists), Wallas (1926) constructed a four-stage macro-model. The creative process begins with a preparation phase of the task consisting of raising issues, learning, and collecting information. The creative person focuses on a problem and gathers the relevant elements. The second stage is incubation, which involves unconscious processing. This stage is the least visible one; interviews with eminent creators (e.g., Poincaré 1908) suggest that this phase involves associative thinking yielding potential solutions to the problem. This period ends with an insight when any relevant novel ideas become conscious. However, one or several solutions may remain vague and not obvious at this stage. The process of creation ends with a verification phase. This refers to a sub-process of evaluation in which ideas are developed into a definitive form. Patrick (1935, 1937, 1938) observed that each stage may overlap with others. She found, for example, that an artist could incubate during preparation or verify the idea before starting to sketch. Other researchers have investigated specific aspects of the creative process (Busse and Mansfield 1980; Cagle 1985; Goswami 1996; Hadamard 1945; Ochse 1990; Osborn 1953; Taylor et al. 1974). For example, Mumford et al. (1991) specified the process of reorganizing information involved in insight, and Mumford et al. (1994) differentiated problem-finding (detecting that something is not right, not good, or lacking), problem-posing (formulating the problem) and problem construction (proposing a description of the problem).

Recently, Botella and Lubart (2015) described the dynamic of the creative processes comparing students in three domains: art, design, and science-engineering. Even if the stages are just as much as cited by students of the three domains, authors showed, for example, that science-engineering students referred more to insight and associative thinking and less to implementation and breaks than art students; and science-engineering students referred more to consideration of constraints and benefit from chance and less to documentation, finalization, and divergent thinking than design students.

**Figure 1:**
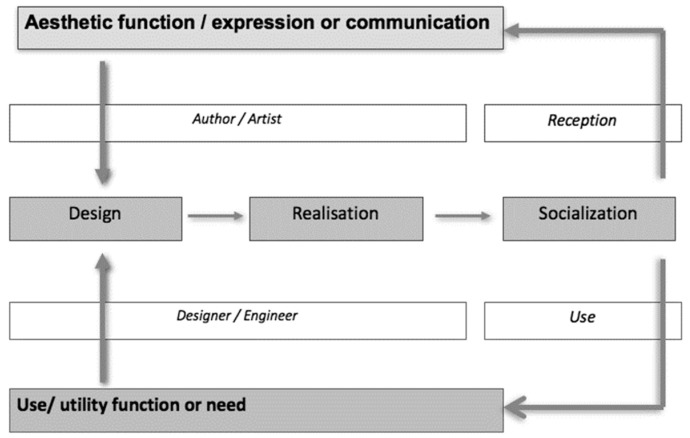
The theoretical “design–realization–socialization” model (Didier and Leuba 2011).

In the context of design, the theoretical “design–realization–socialization” model is conceived initially as a means of mapping the creation process during the manufacture of an object or a project (Didier and Leuba 2011, see Figure 1).

This model suggests two discrete professional approaches: the approach of the author/artist, who creates an object with an aesthetic function corresponding to an expression or communication; and the approach of the designer/engineer, who designs an object with a utility function intended to satisfy a purpose or need (Didier and Leuba 2011).

In this model, the activity of design demands identifying and analyzing the problem and finding innovative and appropriate situations for realization (Bonnardel and Didier 2016). The activity of design includes different stages of the creative process such as definition, reflection, structuration of ideas, dealing with constraints, documenting, and taking decisions (Bonnardel 2006). During these stages, many cognitive factors are used such as divergent thinking, convergent thinking, and associative thinking to generate a hypothesis, evaluate solutions, and make appropriate decisions (Bonnardel and Didier 2016). The design phase appears as a key element where the designer/engineer must abandon everyday ideas to explore the world of ideas and to propose innovative solutions (Didier and Bonnardel 2017). Divergent thinking, underused in schools (Lubart et al. 2015), is one of the key phases of the design activity (Didier and Bonnardel 2017). The selection of the ultimate idea must then factor in all the needs and constraints of the object. This demands convergent thinking that takes into account the individual’s different parameters. The task of innovation, combined with the constraints imposed by materials, as well as the implementation and functional use of the object, stimulates pupils and systematically teaches them to anticipate (Didier and Leuba 2011). In a design education context, Sawyer (2022) has observed that the object is central to the interaction between student and teacher. These exchanges lead to an iterative and ambiguous creative process through the exploration and emergence of ideas by students.

More generally, current macro-models diverge somewhat on the nature of the creative process, and almost all of them are focused on the cognitive factors involved in the creative process, without taking into account the other factors—notably the conative, emotional, and environmental factors—that are involved. Yet, combining these four sets of factors—i.e., following a multivariate approach—makes it possible to draw a more complete picture of the creator and the creative process. Recent conceptions of the creative process described it as a dynamic phenomenon in which it is possible to skip a stage, to return to a previous stage, to iterate a stage (Tayal 2013), all in association with multivariate factors (Botella and Lubart 2015). Thus, the creative process would be not only a linear succession of cognitive stages, but should involve a combination of emotional, environmental, and motivational abilities.

At this point of the state of art, it is important to note that an approach describing the micro-processes instead of macro-process is, in the present study, considered included in the stages of the macro-process approach (Botella et al. 2016) which, being more global, allows us to explore the sequence of the stages as well as the stages themselves.

### 1.2. The Multivariate Approach

According to the multivariate approach, the creative person is defined as possessing a combination of cognitive, conative, and emotional components associated with favorable environmental conditions (Amabile 1996; Gardner 1993; Lubart 1999; Lubart et al. 2015; Sternberg and Lubart 1991, 1995). The cognitive component corresponds to the intellectual abilities involved in creativity. Lubart et al. (2015) proposed a summary of cognitive capacities including synthetic capacities of identification, definition, and redefinition of the problem, selective encoding (to encode only relevant information for solving the problem), selective comparison (to observe similarities between various domains), selective combination (associations between the ideas collected). Several researchers have proposed an elaboration–evaluation cycle in which ideas are perpetually generated and judged (see Bonnardel 1999). The conative component concerns personality traits and motivation. Creative individuals are usually described as open to new experiences (Feist 1998; Furnham and Bachtiar 2008; Gough 1979; McCrae and Costa 1987; Wolfradt and Pretz 2001; Zenasni et al. 2008). Openness is reflected in a dynamic fantasy life, aesthetic sensibility, emotional awareness, need for originality, intellectual curiosity, and a strong personal value system (Helson 1999). Creative people are also tolerant of ambiguity (Barron and Harrington 1981; Levy and Langer 1999; Sternberg and Lubart 1995; Tegano 1990; Zenasni and Lubart 2001, 2008). Moreover, past experiences in creativity contribute to the creative self-beliefs as a conative factor (Myszkowski et al. 2022).

The emotional component of the multivariate approach corresponds to emotional states and traits (Botella et al. 2011; Zenasni and Lubart 2008). For example, emotional traits positively correlated with creative performance are emotional clarity (George and Zhou 2002) and emotional intelligence (Wolfradt et al. 2002). In a meta-analysis of 62 empirical studies, Davis (2009) confirmed that positive emotional states promote a playful approach to the task increasing creative performance. Finally, the multivariate approach emphasizes the environment which offers physical and/or social stimulations and can help the generation and maturation of ideas, thus reinforcing motivation (Lubart 1999). For example, multicultural experiences are linked to creativity (Fürst and Grin 2021). The environment includes the appreciation of creativity through social judgment. For Sternberg and Lubart (1995), creativity involves more than a sum of all these components: certain constituents can partially compensate each other. For example, a strong degree of motivation can mitigate a lack of knowledge. These components interact among themselves; the combination of high intelligence and strong motivation may enhance creative performance in a multiplicative manner. Thus, the multivariate approach focuses attention on the various constituents involved in artistic creative activity and aims to examine the interactions between them.

### 1.3. Teaching Creativity in the School Context

Concerning the development of creativity in school, the work of Miller and Dumford (2014) shows that the creative process is connected to other transversal capacities. The development of creative thinking at school increases school performance (Sternberg 2003).

To develop creative abilities of the students, the 1999 NACCE report (NACCE 1999) sets up three main principles for the teachers to fulfill: “(1) encouraging students to believe in their creative potential, (2) identifying students own creative strengths in different areas, (3) fostering the creative potential of all children and realize that the best way to enhance creativity and is through the process of being creative” (Berggraf Saebø et al. 2007, p. 210). Craft (2005) introduced a fourth principle to adopt a learner-inclusive approach to pedagogy in which the students can co-participate or co-create with each other or their teacher. The teacher’s creativity is therefore essential in order to free the students’ creativity (Massie et al. 2022).

The conditions to teach creativity with creative learning for Lucas (2001) are based on four conditions: (1) the need to be challenged, (2) the elimination of negative stress, (3) feedback, and (4) the capacity to live with uncertainty. According to Birch et al. (2017), different creative and concrete approaches are already tested in the classroom with children in the United Kingdom. In fact, in collaboration with architects and designers, these concrete approaches enhance creativity with the use of “creative posture” such as designer or architect for the children during creative concrete projects (Birch et al. 2017).

In another school context, especially in France, different alternative pedagogies had already been tested in diverse learning environments, such as Montessori, Steiner, and Freinet to foster the creativity of pupils (Besançon and Lubart 2008). These schools improve the creativity and motivation of children by using a pragmatic approach, directly related to daily life by using and producing objects during learning (Besançon and Lubart 2008; Besançon et al. 2015).

### 1.4. The Creative and Manual Activities Context

Following this aim to assess pupils on their ability to solve unusual, nonroutine problems whilst relying on their comprehension of techniques about everyday life (so to be creative and to mobilize multivariate factors), the present research will take place during Creative and Manual Activities, which corresponds in Switzerland to a discipline taught with pupils aged from 6 to 15 years. The relation to practice intrinsic to this discipline is characterized by the crafting of functional objects aimed at the acquisition of dexterity, precision, rigor, and skill.

The Creative and Manual Activities also intended to foster the development of creativity in pupils involved in solving complex problems. These complex problems rely both on discipline-specific knowledge and cross-domain skills (communication, creative thought, collaboration, learning strategies, and reflective approach) in the development of the creative process. The pedagogical approach of Creative and Manual Activities aims to train pupils to solve concrete problems as a designer or as an engineer. In fact, in the compulsory schooling of the Creative and Manual Activities, pupils learn how to design and to realize a technical object or a technical system with a use-function intended to satisfy a purpose or a need (Didier and Leuba 2011). This pedagogical approach encourages pupils to reflect on the knowledge of materials, anticipation, planning the work to be performed, and on the constraints to the use and/or reception of the object (Didier and Leuba 2011). These cognitive operations gradually lead pupils or students to resolve complex situations, to become autonomous by abandoning the posture of mere executor, and to solve problems (Didier 2016). In this aim, Massy and Perrin (2022) introduced in a class of 9–10-year-old students in ACM a “workshop notebook” allowing the pupils to have a space to think about their approach and thus reinforce the learning of design. The discipline of Creative and Manuals Activities taught in the compulsory schooling uses a didactical approach based on the theoretical model “Design–Realization–Socialization” (DRS; Didier and Leuba 2011; Didier 2017) to develop the creativity of pupils. To teach creativity, this pedagogical approach puts students in an artistic or designer posture during the creative process (Didier 2016). Adopting the DRS model during the Creative and Manual Activities in the specific context of compulsory schooling encourages pupils’ reflection on the knowledge of materials, anticipation, planning the work to be performed, and on the constraints of the use and/or reception of the object (Didier and Leuba 2011). The cognitive operations induced by the activity of design (Bonnardel 2006) lead pupils to enter into a contextualized creative process (Didier and Bonnardel 2017). The designer approach developed by the Creative and Manual Activities taught with the DRS model trains the pupils to anticipate and to create a hypothesis that will be tested and confronted in the concrete world with tests and manipulations. The DRS model has to be taught with a skills-based pedagogy which is used to solve concrete complex problems. This kind of pedagogy mobilizes different conative capacities such as perseverance, risk-taking, willingness, autonomy, and intuition. The skills-based pedagogy involves conative components of the learning process (Rogiers 2001). This pedagogy trains also the ability of the learner to control negative emotions such as fear, satisfaction, doubts, frustration, surprise, sadness, and anger when they are confronted with concrete challenges with complex tasks.

### 1.5. Aims of the Present Study

The main objective of this research is to describe a creative process and the factors that influence it in a real context of creativity learning. Introducing analytical, creative, and practical activities (Sternberg and Grigorenko 2004) during the manufacturing of technical objects remains a process that is underused in the context of schooling. Because of this, it has become indispensable to better understand the workings of applied creativity, which could be developed in all pupils taking part in obligatory schooling. Activities involving the design and realization of utility objects allow us to reinforce motivation and the sense of learning in pupils. We will thus be able to better access the stages of evaluating solutions, the decision-making processes, and the iterative processes that require tracing one’s steps back to reformulate a problem. These aspects, related to problem-solving, are directly highlighted by the PISA inquiries (OECD 2014), which note the weakness with which pupils can solve technical problems that require the deployment of innovative and suitable ideas. 

This study consisted of observing the development of creativity in specialized workshops for the development of creativity: a classroom designed for creative and manual activities in compulsory schooling. Davies et al. (2013) pointed out the effects of architecture and the psychosocial and psychological characteristics of the environment in the development of creativity. Longitudinal self-observations of the creative process of pupils will allow us to set theoretical bases relative to how the creative process unfolds. Indeed, the construction and analysis of data collection on creative processes will lead to models of the creative process of students in primary schools. An additional goal is to observe the creative process of pupils in situ, that is, as it takes place and in the context in which it occurs naturally. 

In the context of this study, we focus on the teaching of the engineering approach where the pupils were asked to design and to realize a technical object, e.g., a fountain project. The objective is therefore to describe the implementation of a creative process in this particular educational context. Taking place in a specific context, this study is exploratory. We will explore how the stages of the creative process and the multivariate factors shift over the course of a project in creative and manual activities; we will then propose a model of the creative process and the multivariate factors in this context of creativity learning. We expect that each lesson in the project will mobilize different steps and multivariate factors.

## 2. Materials and Methods

### 2.1. Participants and Data Collection

The sample was composed of a whole class of 22 pupils in primary schooling in the French part of Switzerland during Creative and Manual Activities lessons (M = 11.38 years, SD = 0.65, range = 11–13), mostly females (67%). All parents consented to this study and since school is compulsory, there is no experimental mortality. It should be noted, however, that a pupil may be exceptionally absent from a lesson (for example, if he or she is sick) that occurred once to two pupils during the project.

### 2.2. Materials

To model the creative process of each participant by identifying the stages they go through to create an object, the transitions between these stages, and the multivariate factors involved in these stages, we used a Creative process Report Diary (CRD, Botella et al. 2017). This CRD methodology is situated in an activity-centered ergonomic approach (Daniellou and Rabardel 2005) which considers that activity cannot be understood only on the basis of observation by a third-party analyst. Instead, it is necessary to rely on personal narratives and descriptions of daily experiences by the participants themselves.

The longitudinal self-evaluations consist of having pupils indicate the stages and multivariate factors they mobilized during the session. With these CRD, we will obtain important information regarding the individual course of the creative process. Because it is difficult to capture the unconscious level of the creative process with self-report, the CRD method offers a manner to capture the activities realized (easy to answer for participants) and these activities reveal unconscious processes. For example, the stage of “break” (conscious) corresponds to the incubation phase (unconscious).

Adapting from previous research studies listing the stages of the creative process (Botella et al. 2013; Botella et al. 2011; Glăveanu et al. 2013) to pupils, 14 stages of the creative process are considered: definition of the problem, reflection, documentation, consideration of constraints, insight, associative thinking, divergent thinking, convergent thinking, the benefit from chance, implementation, finalization, judgment, taking a break, and abandonment; as well 20 multivariate factors: perseverance, structure, patience, perfectionism, willing in work, risk-taking, optimism, autonomy, intuition, knowledge, ask for help, communication with others, teamwork, fear satisfaction, doubts, frustration, surprise, sadness, and anger. In this children’s version of the CRD, the stages and factors are presented by a sentence and a picture. These images have been specially designed for children (Botella et al. 2019).

The advantage of this method of CRD and analysis lies in the fact that participants do not need to be aware of their creative process so that we can model it. The pupil is only asked to mark what he or she did during a session (the stages realized and the multivariate factors experienced) without rebuilding the whole structure of the process.

### 2.3. Procedure

In the context of compulsory schooling, pupils have the Creative and Manual Activities lessons every week for two sessions of 45 min per week through the half-year. During this course, pupils perform successive projects. The study was implemented as a project on the creation of a fountain during 5 lessons. Before the beginning of the fountain project, the pupils watched a movie with their teacher about artistic fountains created by the French artist Jean Tinguely, to enhance their inspiration and their creativity. The teacher^1^ also introduced the CRD to the pupils and each vignette (stages and factors) was discussed in class and explained. For example, the “abandonment” stage corresponds to giving up an idea, not the whole project. During their Creative and Manual Activities course, the fountain project has been taught during 5 lessons of 90 min each, focused on collective and individual productions. 

**Figure 2:**
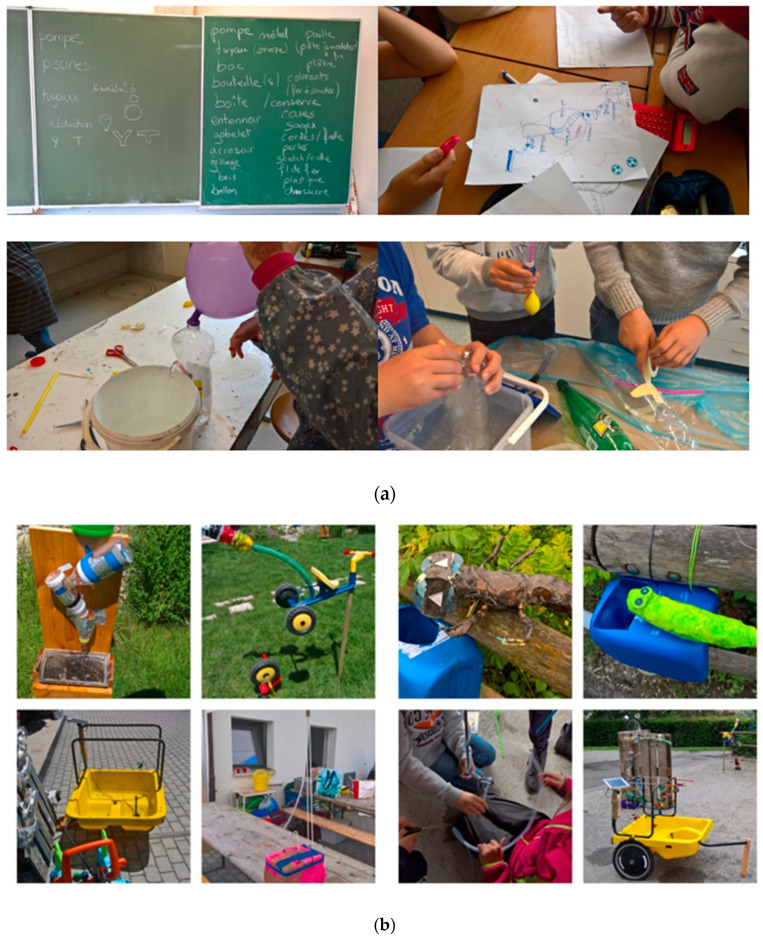

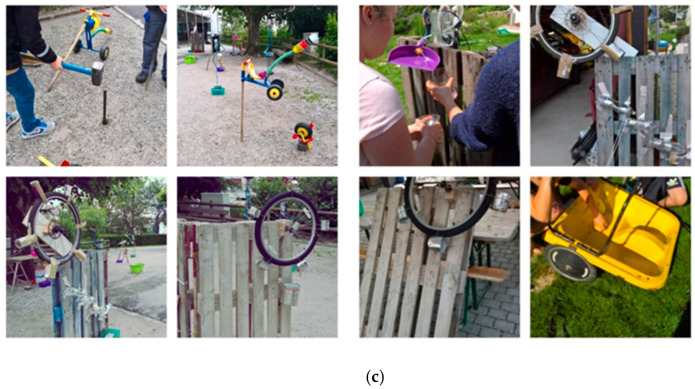
(**a**) Research of fountain project (design and realization of fountains with recycled materials). (**b**) Research and experimentations of pupils during the design stage. (**c**) Fountains made by pupils during the Creative and Manual Activities in the compulsory schooling.

Concerning the organizational structure of the 5 lessons, the first and the second lessons were focused on research and design of the fountain project by the pupils (see Figure 2a). Different creative design activities have been developed within the team of teacher and pupils such as identifying problems, structuring complex tasks without predefined procedures, communicating ideas, creating a hypothesis, and experimenting with them to evaluate the funding solutions. So, the first lesson was focused on research concerning fountains, and the second lesson was dedicated to the design of the fountain project.

The third lesson was centered on the production of the projects. Pupils had the opportunity to test their hypothesis, to manipulate and to use different materials, and to learn in a concrete environment, technological and scientific learning, such as movement, solidity, and weight of materials. The third lesson was centered on the pragmatical approach of teaching how to separate, to transform, and to fix different recycled objects and materials (see Figure 2b). The fourth lesson has been used to fix and to assemble the different constructions of the pupils. The fifth and last lesson was devoted to the installation and the exhibition of the different fountains (see Figure 2c).

In order not to interrupt the creative activity, the CRD is completed only once at the end of the lesson in which pupils were invited to report the stage(s) they did during the lesson, and the multivariate factor(s) they experienced. So, in each session, the pupil could mark from 1 to 14 stages of the creative process and from 1 to 20 multivariate factors. The responses were then 0 (stage/factor was not checked) or 1 (stage/step was checked). On average, pupils checked 5.28 stages (SD = 1.51, between 2.2 and 8.2) and 8.26 factors per session (SD = 2.65, between 3 and 12.2).

## 3. Results

Analysis of CRD is quantitatively descriptive. The analysis will allow us to identify which factor(s) has(ve) been mobilized by each participant at each phase of the creative process during each evaluation. First, we will examine the dynamics of the stages of the creative process. At each lesson, the frequency of pupils marking a stage will be reported^2^. Then, the same will be done for the multivariate factors involved in each lesson. Lastly, based on a correspondence analysis, the dynamic of the stages and the multivariate factors will be integrated into a modelization of the creative process. This statistic was recommended to analyze the CRD (Botella et al. 2017) and used in previous research with students (Botella and Lubart 2015) as well as with pupils (Didier et al. 2022).

### 3.1. Dynamic of the Stages

#### 3.1.1. Variations of the Stages

**Figure 3:**
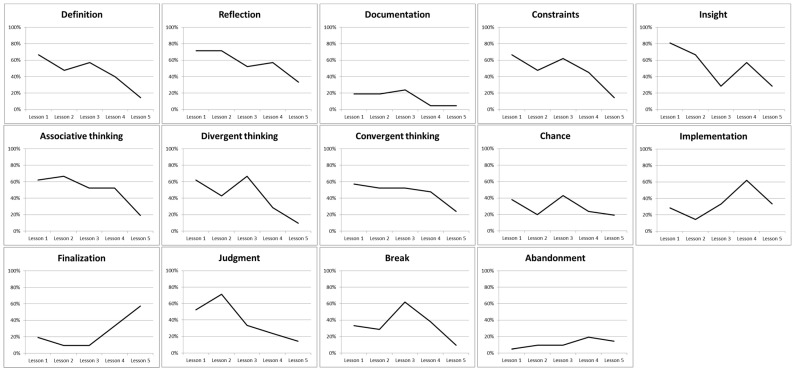
The stages of the creative process during the 5 lessons of the fountain project.

In Figure 3, the variations of the frequency of the pupils reporting each stage at each lesson are indicated. From this figure, we can see that stages of documentation, chance, and abandonment are not frequently reported during all times of the project. We also saw that the frequency of stages of definition, reflection, consideration of the constraints, associative and convergent thinking decreased, more or less quickly, over the lessons. Implementation and finalization increased slowly over the lessons. Finally, other stages had specific variations as insight, which started by being very reported by pupils and then decreased until lesson 3 to increase again in lesson 4 and decreased in lesson 5. This variation indicated that lesson 3 was less insightful for pupils but they had an idea in lesson 4. In the same vein, pupils declared less divergent thinking in lesson 2 but more in lesson 3. The stage of judgment increased a little between lessons 1 and 2 and then decreased mainly. It is also important to note that the frequency of 100% was never mentioned, indicating that not all the pupils in the class did the same stage(s) in a lesson. So, even if the project is the same, this result showed inter-individual differences in the progress of the creative process.

#### 3.1.2. Mobilization of the Stages

During lessons 1 and 2, focused on the research and the design parts of the project, over 50% of the pupils mobilized the stages of reflection, constraints, insight, associative thinking, convergent thinking, and judgment. These different stages are used to solve complex tasks without predefined procedures. The research and design activities worked on during lesson 1 and 2 are essential to generate creative and innovative ideas adapted to the context. The divergent thinking has been frequently mobilized during lessons 1 (61.90%) and 3 (66.67%), but interestingly less in lesson 2 (42.56%), in which pupils had to design their project. 

Over 50% of the pupils also mobilized the constraints and judgment stages during lessons 1 and 2, suggesting an important use of these transversal skills during different stages of the problem-solving of the fountain project. In lesson 4 in which pupils were invited to fix and assemble the elements of their fountain, a majority of them mobilized stages of reflection (57.14%), insight (57.14%), associative thinking (52.38%), and realization (61.90%). It is interesting to observe that the last lesson mobilized mainly, but not exclusively, to finish the project (57.14%), whereas 19.05% of pupils marked to abandon an idea.

### 3.2. Dynamic of the Multivariate Factors

#### 3.2.1. Variations of the Factors

**Figure 4:**
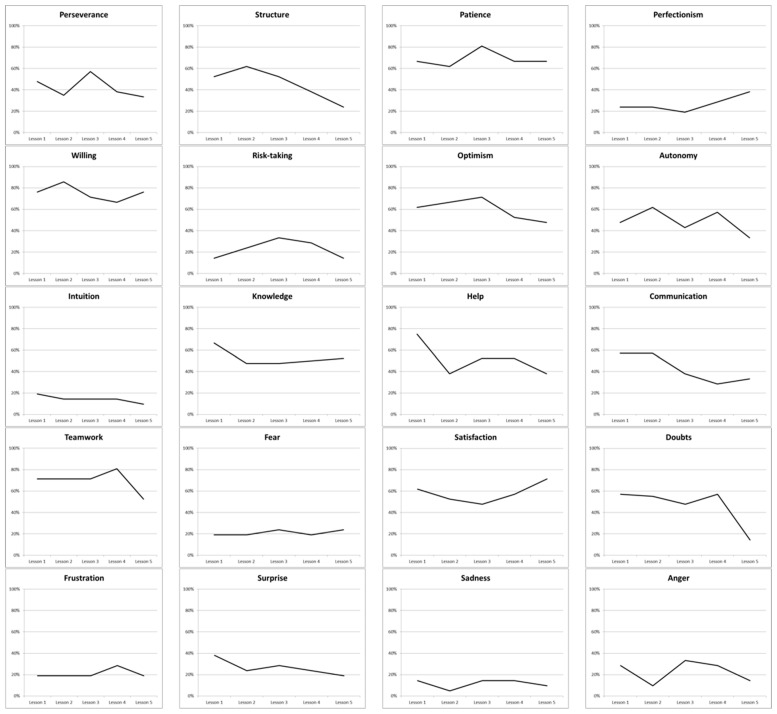
The multivariate factors involved in the creative process during the 5 lessons of the fountain project.

In Figure 4, the variations of the frequency of the pupils reporting each multivariate factor at each lesson are indicated. From this figure, we can see that stages of perfectionism, risk-taking, intuition, fear, surprise, sadness, and anger are less reported, whereas others are patience and willingness. Some multivariate factors decreased only for the last lesson as teamwork and doubts, whereas perfectionism increased only at this last lesson. It is interesting to see that something may have happened in lesson 3 in which perseverance, patience, risk-taking, and optimism increased slightly, whereas autonomy and satisfaction decreased slightly too.

#### 3.2.2. Mobilization of the Factors

Cognitive, conative, and emotional abilities were also concretely used in this project. Over 50% of the pupils used conative factors such as structure, patience, willingness, optimism during lessons 1, 2, and 3. A majority of pupils used the autonomy factor in lessons 1 (48%), 2 (62%), and 4 (57%). The knowledge factor indicates an overall percentage from 48% to 67% during the lessons. Another important skill used during the research and design process (lessons 1 and 2) was communication (57% in both lessons). The teamwork and satisfaction multivariate factors have been mobilized during all lessons. Concerning Doubts factor, the majority of pupils utilized this factor from lessons 1 to 4.

### 3.3. Modelization of the Creative Process

**Figure 5:**
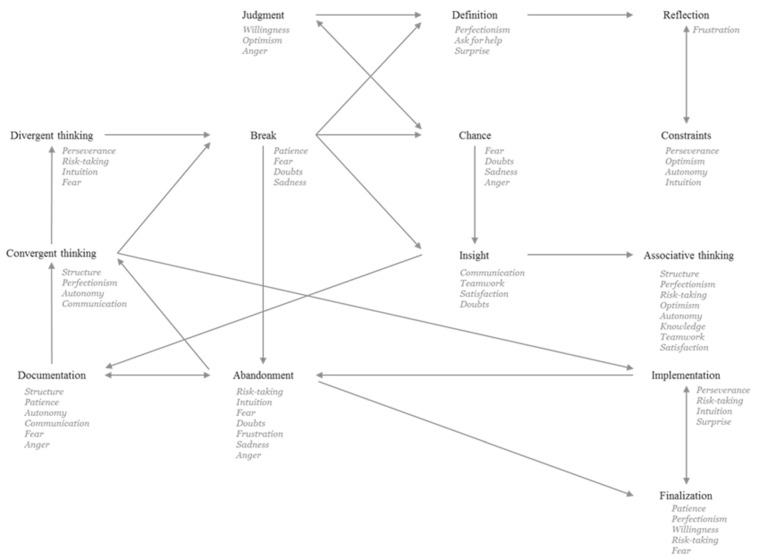
Graphical representation of the transitions between the stages of the creative process and the multivariate factors associated with each stage.

First, for each pupil, a transition table was built to find out at what stage he/she started to move to another stage. This transition table includes as many rows and columns as stages of the process (i.e., 14 steps). Each cell of the transition table counts the number of times a participant checked, for example, the Reflection stage after the Definition stage. Second, a global transition table was calculated for the whole class, summing all the transition tables of the pupils. Finally, a correspondence analysis was run on the global transition table allowing to identify the sequence of the stages of the creative process. A similar table was constructed to examine the link between the stages and the multivariate factors and then, another correspondence analysis was lead. Results are graphically represented in Figure 5. Arrows represent the most frequent transitions between the stages, and the text below the name of a stage corresponds to the multivariate factors mainly involved in this stage compared to other factors and stages. Note that all transitions between stages are possible and that all factors have been associated at least once with each stage. Figure 5 then presents only the most salient transitions and profiles.

A global look at Figure 5 shows that the creative process of the pupils creating a water fountain is not linear. Several interactions (double arrows) appear between the stages: chance–evaluation, questioning–constraints, documentation–abandonment, and realization–finishing. Many interesting transitions are observed. Evaluation of the project leads the pupils to define and then to question it. Breaks are taken after experimentation or organization stages and lead to defining the project, chance, insight, or abandonment. Chance also conducts at insight and insight leads to documentation or associated ideas. Abandonment and documentation can loop on the organization phase that leads to experimentation or realization. Realization leads some pupils to abandon ideas, but abandonment can also conduct to finish the project.

The multivariate profile associated with each stage of the process indicates that definition involves perfectionism, ask for help, and surprise. When pupils experiment with ideas, they mobilize perseverance, risk-taking, intuition and they feel fear. The insight stage is associated with communication with others, teamwork, satisfaction but also some doubts. Taking the chance into account is linked to many negative feelings (fear, doubts, sadness, anger), probably because pupils cannot manage this stage. The abandonment phase is also associated with negative feelings (fear, frustration, doubts, sadness, anger), risk-taking, and intuition. Finishing a project involves patience, perfectionism, willingness, risk-taking, and fear.

## 4. Discussion

In this study, we examined the development of creativity of pupils in Manual and Creative Activities through their creative process in light of the multivariate factors. We showed that the stages and the multivariate factors vary across the duration of the fountain project.

### 4.1. Mobilization and Variations of the Stages of the Creative Process

The creative process of pupils started with the stages of reflection, constraints, insight, associative thinking, convergent thinking, and judgment, whereas it finished mainly with the finalization stage. It is interesting to observe that lesson 4 involves also reflection, insight, associative thinking, and realization, showing that reflection, insight, and associative thinking are key stages during all the process. Mumford and collaborators (1991) had already explained that insight involves reorganizing the information, that is, with reflection and associative thinking, important to generate an idea. Linked to the judgment phase, this result corresponds to the elaboration–evaluation cycle, in which ideas are perpetually generated and judged (see Bonnardel 1999).

For Bonnardel and Didier (2016), in design, it is important to abandon the everyday to explore the world of ideas and to propose innovative solutions. However, in the present study, pupils had rarely declared they abandoned an idea. Maybe this result could be due to the age of the pupils, who never give up until the end of the project, and more especially because of the schooling context. In the same vein, the perseverance of the pupils could explain also why the stage of the benefit from the chance was not reported. Surprisingly, the stage of documentation was also not reported. This result could be explained by the fact that the teacher had already shown a movie about artistic fountains to pupils to enhance their inspiration and their creativity.

### 4.2. The Creative Process of Pupils in the Posture of Designer/Engineer

As Miller and Dumford (2014) underlined the involvement of transversal capacities in the creative process, pupils mobilized a similar design process as the designer/engineer. During the design-solving problem, the designer or apprentice designer (in our context pupils) mobilizes complex cognitive processes such as the creation of hypotheses, analyses, synthesis, evaluation of ideas, convergent, divergent and flexibility thinking, dealing with constraints and taking decisions (Bonnardel 2006).

According to our observations, pupils have used adapted skills to appropriate a posture of designer/engineer in this project helping them to solve complex problems without predefined procedures. This pedagogical fountain project permits the development of creative teaching for children, encouraging them: (1) to believe in their creative process, (2) to identify their creative strengths such as autonomy, optimism, and knowledge. The Design–Realization–Socialization model used in this pedagogical approach seems to be appropriate for pupils to discover a way to enhance creativity through the process of being creative and to adopt an inclusive approach to pedagogy (Craft 2005). Pupils had to co-participate and they had to co-create with each other and their teacher.

It is interesting to note that some of the present findings were also already observed in the scientific-engineering creative process of students (Botella and Lubart 2015): transition from definition to reflection, the interaction between reflection and consideration of the constraints stages, transitions from insight to associative thinking, from associative thinking to convergent thinking, from convergent thinking to take a break, from the benefit from chance stage to finalization, and from the break to benefit from chance. Some other transitions were observed in the design creative process as the one from insight to documentation (also observed in the artistic creative process), from convergent thinking to implementation, and the interaction between implementation and finalization (also observed in the artistic process). Finally, an additional transition was only similar to the artistic creative process such as the transition from judgment to the benefit from chance. Other transitions found in the present research are specific to the context. These similarities with the study of Botella and Lubart (2015) suggest that the process of pupils creating a water fountain in their Manual and Creative Activities is close to one developed by science-engineering or design students. That fits the main purpose of this lesson, the objective of which is to position the pupils in a designer/engineer role.

### 4.3. Mobilization of Multivariate Factors during the Creative Process

Concerning the mobilization of multivariate factors, the involvement of autonomy (lessons 1, 2, and 4) is encouraging, suggesting appropriate conditions in the learning of a complex task. Additionally, the mobilization of the knowledge factor indicates that the skill-based pedagogy trains the students to integrate knowledge in different complex situations (Rogiers 2001) and the ability to learn how to use and to mobilize knowledge in unknown situations. The involvement of communication and teamwork points to an adapted way to enhance collaborative teamwork in concrete, collaborative, and challenging school contexts, such as the fountain project. Teamwork is a transversal capacity considered also as a key condition to foster teaching creativity and creative learning (Lucas 2001). The satisfaction experienced by pupils through the entire project indicates that positive emotions are adapted to support creativity (Davis 2009; Lubart et al. 2015). Concerning the Doubts factor, this mobilization during the fourth first lessons suggests that pupils had to be tolerant to ambiguity (Barron and Harrington 1981; Levy and Langer 1999; Sternberg and Lubart 1995; Tegano 1990; Zenasni and Lubart 2001, 2008). The key conditions proposed by Lucas (2001) for teaching creativity and creative learning are the need to be challenged; the elimination of negative stress seems to be present in this pedagogical approach by the fact that negative emotions such as fear, frustration, sadness, and anger appear in under 33% of the pupils in all lessons.

### 4.4. Limitations

A first limitation of this study is that it was conducted in the French part of Switzerland. However, it should be remembered that Switzerland is divided into cantons, each of which has its own functioning. Thus, the results of this study are not generalizable to other cantons and more extensively to other contexts. However, despite this limitation in the generalization of results, this study provided an opportunity to explore a specific field of creativity training that can be transferred to other contexts.

Moreover, because the study of Botella and Lubart (2015) described specific students (design or engineering students) creating a specific product (a poster for a conference or a functional kitchen located in a campervan) in a specific context (10 sessions over 8 weeks of the workshop) as in the present study (pupils in manual and creative activities, creating a water fountain during 5 lessons), the results are not completely overlapping. This kind of self-observation had to be replicated to find solid similarities or differences between the studies.

However, despite these limitations, the present study offers support that the position of designer/engineer asked by the teacher is respect by pupils and helped them to create. The differences between pupils and science-engineer students could be a way for improving the teaching. For example, it is interesting to find that divergent thinking leads to taking a break (considered as the incubation stage).

Finally, this study is limited by the sample size (22 students), which here represents the entire class. Since different projects cannot be aggregated into one study, it will be important to replicate these results in future research.

### 4.5. For Futher Research

The CRD propose a good pedagogical support to improve the creative metacognition of the pupils. Kaufman and Beghetto (2013) defined the creative metacognition as “a combination of creative self-knowledge (knowing one’s creative strengths and limitations, both within a domain and as a general trait) and contextual knowledge (knowing when, where, how, and why to be creative)” (p. 160). Moreover, the authors proposed that teachers help students to better understand what creativity is (new and adapted; the difference between levels of creativity), what are the benefits and the costs of creativity (to manage the risk-taking), what are their strengths and limitations (the teacher had to provide adequate feedback to maintain the attention and motivation of students), and to recognize the expression of creativity. The CRD used in the present study could be pedagogical support to enhance the metacognition of pupils on their creative process and thus, on a complementary point proposed by Kaufman and Beghetto (2013). Other educational projects may therefore use the CRDs for teaching creativity or in a larger teaching context, and future research can therefore more fully explore the link between metacognition and the use of CRD in the classroom. In the same vein, beliefs in one’s creative abilities could be a focus of future research. Indeed, there are links between creative metacognition, creative self-beliefs and creative performance (Anderson and Haney 2021).

In future research, other creative projects could be investigated, varying as much in terms of content, the field concerned, the duration, and the pedagogical organization of the class group. Once extensive replications have been carried out, a meta-analysis will allow for the emergence of a creative process specific to pupils in a creativity learning context. This point seems to us to be a major challenge in order to distinguish between models built from experts in different fields (designers, scientists, artists, etc.) and pupils in learning. This model will be of significant use to teachers in the real world who will then have a better vision of what their pupils are actually learning.

## 5. Conclusions

Finally, as recommended by the PISA inquiries, the training of creativity in school is the lever to improve school performance (Sternberg 2003) and, more globally, a major asset for our society. The DRS model applied in the Creative and Manual Activities supports a pedagogy in which the students can co-participate or co-create with each other or their teacher (Craft 2005). The present study proposes a beginning of pedagogical observation to better train pupils to solve complex problems without predefined procedures, which fits the enhancing of creativity in compulsory schooling which appears as the next fundamental challenge for teachers (OECD 2014).

Based on previous studies with creativity experts that have described the creative process specifically in the field of design and identified the multivariate factors that influence it, and also on work conducted in the field of creativity education and training, particularly in the context of creative and manual activities, this exploratory study was implemented with a class of 22 pupils during the realization of a water fountain project. Following their activity through the Creative Process report Diary methodology allowed us to highlight changes in process stages and multivariate factors between the 5 lessons of the project. Through correspondence analysis, we were able to represent transitions between creative process stages and examine which mutivariate factors were associated with these stages. This study thus provides an illustration of how the creative process actually unfolds for pupils in a creativity learning context. It has implications both for education in teaching creativity and for more fundamental research on the pathway of the creative process.

## Data Availability

The data presented in this study are available on request from the corresponding author.

## Notes

1 The teacher is experienced and has been using CRD for many years in her classes.

2 Frequency was used rather than the number of pupils to compensate for their absence from a session.
